# Cut-off Values of the Respiratory Muscle Power and Peak Cough Flow in Post-Stroke Dysphagia

**DOI:** 10.3390/medicina56120635

**Published:** 2020-11-24

**Authors:** Yeon Jae Han, Jungjae Lee, Dong Gyun Sohn, Geun-Young Park, Youngkook Kim, Hae-Yeon Park, Sang-A Jung, Sun Im

**Affiliations:** 1 Department of Rehabilitation Medicine, Bucheon St. Mary’s Hospital, College of Medicine, The Catholic University of Korea, Seoul 14647, Korea; duswohan@gmail.com (Y.J.H.); slowhand1986@gmail.com (D.G.S.); rootpmr@catholic.ac.kr (G.-Y.P.); bd0495@naver.com (S.-A.J.); 2 Department of Rehabilitation Medicine, Seoul St. Mary’s Hospital, College of Medicine, The Catholic University of Korea, Seoul 06591, Korea; blue.joanarc@gmail.com (J.L.); hy2park@naver.com (H.-Y.P.); 3 Department of Rehabilitation Medicine, Yeouido St. Mary’s Hospital, College of Medicine, The Catholic University of Korea, Seoul 07345, Korea; england2formac@gmail.com

**Keywords:** cough, deglutition disorder, stroke, diagnosis, inspiration, expiration

## Abstract

*Background and objectives:* This study aimed to determine the cut-off values of the following three respiratory pressure meters; the voluntary peak cough flow (PCF), maximal expiratory pressure (MEP) and maximal inspiratory pressure (MIP); associated with post-stroke dysphagia and assess which of these parameters show good diagnostic properties associated with post-stroke dysphagia. *Materials and Methods:* Retrospective analysis of a prospectively maintained database. Records of patients with first-ever diagnosed dysphagia attributable to cerebrovascular disease, who had performed spirometry measurements for the PCF, MIP and MEP. *Results:* From a total of 237 stroke patients, 163 patients were diagnosed with dysphagia. Those with dysphagia had significantly lower PCF values than those without dysphagia (116.3 ± 75.3 vs. 219.4 ± 91.8 L/min, *p* < 0.001). In addition, the former group also had lower MIP (30.5 ± 24.7 vs. 41.6 ± 25.7 cmH_2_O, *p* = 0.0002) and MEP (41.0 ± 27.9 vs. 62.8 ± 32.3 cmH_2_O, *p* < 0.001) values than the latter group. The receiver operating characteristic curve analysis showed that the PCF cut-off value of 151 L/min (area under the receiver operating characteristic curve [AUC] 0.81; sensitivity 72%; specificity 78.8%) was associated with post-stroke dysphagia. The optimum MEP and MIP cut-off were 38 cmH_2_O (AUC 0.70, sensitivity 58%; specificity 77.7%) and 20 cmH_2_O (AUC 0.65, sensitivity 49%; specificity 84%). PCF showed the highest AUC results. Results from the univariate analysis indicated that PCF values of ≤151 L/min increased risk of dysphagia by 9.51-fold (4.96–18.23). Multivariable analysis showed that after controlling of other clinical factor, the PCFs at this cut-off value still showed increased risk of by 4.19 (2.02–83.69) but this was not observed with the MIPs or MEPs. *Conclusions:* Our study has provided cut-off values that are associated with increased risk of dysphagia. Among the three parameters, PCF showed increased association with post-stroke dysphagia.

## 1. Introduction

Stroke patients suffer with hemiparesis, which may also affect the respiratory muscle power. Stroke impairs respiratory muscle strength and cough flow by approximately 50% when compared with non-stroke controls [1]. Decreased inspiratory muscle function manifests as pronounced diaphragm weakness on the hemiplegic side [2]. Decreased expiratory muscle function manifests as abdominal muscle weakness on the hemiplegic side [3]. Patients with dysphagia show even more pronounced weakness of these respiratory muscles [2]. In addition to respiratory muscle weakness, stroke patients are known to exhibit decreased cough strength [4].

Adequate expiratory and inspiratory muscle strength is important for producing a strong cough [5,6]. An efficient coughing process involves the following sequence: inhalation, forced exhalation against a closed glottis, and expiration with the release of air from the lungs. Inspiratory muscle weakness would lead to reduced lung volume at the beginning of cough. Expiratory muscle weakness would lead to reduced intrathoracic pressure. Intrathoracic pressure is needed to produce adequate airflow [7]. In such cases, reduced cough strength may be explained by attenuation of the cough reflexes, altered chest wall kinematics, or direct hemiparesis of the muscles involved in coughing such as the inspiratory or the expiratory muscles [2,3,4,5].

Coughing and swallowing are highly sophisticated and interconnected voluntary and reflexive behaviors that serve to protect the airways during swallowing [6]. An efficient cough is produced by the following sequences: inhalation, forced exhalation against a closed glottis, and expiration with the release of air from the lungs. Glottic closure helps to maintain high intrathoracic pressures which further enhances the cough airstream velocity that may adequately provide high expiratory flow to remove aspirated materials from the airway [7]. Improved glottic closure can also be manifested by higher PCF values [8]. Due to impaired glottic function and due to reduced respiratory muscle force after hemiparesis [2,4], stroke patients exhibit lower coughing force. After a stroke episode, both voluntary and reflexive coughing are downregulated, the probability of aspiration pneumonia is increased [9], and the ability of removing the intratracheal secretions and foreign objects is decreased [10]. Thus, in stroke patients, decreased coughing force may lead to aspiration pneumonia [6]. Moreover, it has also been proven that reduced respiratory muscle force may contribute to the increased incidence of chest infection [11].

Cough has traditionally been a key component of bedside clinical evaluation of swallowing [6]. Daniels et al. [12] proposed that voluntary cough should be assessed during swallowing assessment. Additionally, the presence of an abnormal cough improves the validity of the water swallow test [13]. Impaired voluntary cough is subjectively assessed as weak or absent [14]. Although, adequate voluntary cough is often presented as part of the assessment in post-stroke dysphagia, objective cut-off values that may help define a weak cough post-stroke dysphagia have not been determined yet. Likewise, past attempts have been made to define weak coughs based on cough sound. McGuinness et al. reported that cough sound parameters did not reflect the voluntary cough mechanism well and had limitations in intensity [15]. Wallace et al. reported that the relationship between acoustic intensity and cough effectiveness is unclear due to limited number of observations [16]. Therefore, an objective parameter that may help distinguish those with weak cough is much needed.

Stroke patients with dysphagia exhibit more pronounced respiratory weakness [2]. Although current screening tests assess patients’ ability to cough [14], the degree of respiratory muscle strength is still not included in the formal assessment components for screening stroke patients with dysphagia. Respiratory muscle training has been suggested to improve pharyngeal swallowing [17]. In light of the role of respiratory muscle power in producing a cough, one may hypothesize that respiratory muscle power below a certain level may theoretically be applied to help identify those at increased risk of dysphagia. Therefore, an objective parameter that may help distinguish those with poor respiratory muscle power is as much needed like that for the voluntary cough.

Cough aerodynamics measured using a spirometer can be used to objectively measure the peak cough flow (PCF), maximal inspiratory pressure (MIP), and maximal expiratory pressure (MEP) [18]. MIP and MEP reflect the inspiratory and the expiratory muscle power, respectively.

Thus, the purpose of the present study was to determine the cut-off values of the coughing force (measured by PCF) and respiratory muscle strength (measured by the MIP and MEP) [18] and to determine which of these parameters were associated with an increased risk of post-stroke dysphagia, as confirmed by instrumental assessment tools.

## 2. Materials and Methods

The present study involved a post-hoc analysis of previous data obtained from a cohort of prospectively maintained database of patients who were admitted or referred from the stroke unit to the Department of Rehabilitation Medicine between June 2013 and July 2015, set at a university-affiliated hospital [19]. The study protocols were approved by the local ethics committee (HC17RES10080). Consent was waived due to retrospective nature of the study. Newly diagnosed stroke patients with complete medical records who were suspected to have dysphagia at the initial stroke onset and referred for the instrumental swallowing tests were included. The inclusion criterion for this study mandated that patients were seen within 14 days of stroke onset. The exclusion criteria were as follows: (1) history of dysphagia before admission; (2) prior diseases or procedures (such as surgery or radiotherapy) or medical conditions that could affect swallowing function such as acute pulmonary embolism or myocardial infarction; (3) patients with tracheostomy cannula at the time of assessment; (4) patients who could not use spirometers due to poor conscious state or severe cognitive dysfunction; (5) a previous diagnosis of neurodegenerative disorders that may affect swallowing such as Alzheimer’s disease, dementia, Parkinson’s disease, or multiple systemic atrophy; (6) recurrent strokes, and (7) recent respiratory events with desaturation.

### 2.1. Demographic Variables

Demographic data regarding age, sex, and brain lesion location were retrieved. Other risk factors [6,20] reported to be associated with an increased risk of respiratory infections in stroke patients and the elderly including diabetes, arterial hypertension, asthma, smoking status, chronic obstructive pulmonary disease, hyperlipidemia, coronary artery disease, and atrial fibrillation were recorded.

### 2.2. Dysphagia Assessment

All patients who exhibited or complained of difficulty in swallowing were screened by a doctor. If warranted, presence or absence of dysphagia was confirmed using an instrumental assessment tool via fiberoptic endoscopic evaluation of swallowing (FEES) or videofluoroscopic assessment of swallowing (VFSS) by a certified specialist with more than 10 years of experience. The Functional Oral Intake Scale (FOIS) has very good reliability, validity, and excellent sensitivity to change to objectively determine the range of oral intake of patients with neurogenic dysphagia [21]. A score of 5 or lower is consistent with dysphagia, that requires certain dietary adjustments for normal function [22]. The FOIS was obtained at the VFSS or FEES, which were performed according to standard protocols [23,24] by a clinician with more than 10 years of experience. This clinician was blinded to the results of the respiratory pressure meters. Depth of penetration and aspiration were scored according to the Penetration-Aspiration Scale (PAS) with higher scores indicating severe aspiration [25]. For the PAS, the worst PAS score across all boluses were used for analysis [26]. Patients with instrumentally confirmed FOIS scores ≤5 and PAS score ≥2 were classified as patients with clinical dysphagia or the dys (+) group [21,27]. All medical, clinical, and respiratory pressure parameters were compared between the groups.

### 2.3. Respiratory Pressure Parameters

Voluntary PCF was measured after the patients were asked to perform a quick forceful cough. Before the patients’ voluntary PCF was formally measured, verbal instructions were provided by the clinicians to explain the procedures regarding how to produce a cough on command. Subsequently, the clinician performed a live demonstration in front of the patients regarding how to cough on the portable spirometer. Patients with poor comprehension capabilities were allowed to practice with the clinician a few times before the formal assessment. Voluntary PCF as well as respiratory pressure assessments were performed by two independent physiatrists who were blind to the results of each test and who were not involved in the instrumental assessment of swallowing.

Voluntary PCF was measured after the patients were asked to perform a quick forceful cough on the peak flow meter following the guidelines recommended by the American Thoracic Society/European Respiratory Society [28]. The values were presented as the mean of three highest values from five attempts. MIP and MEP were measured using a respiratory pressure meter (Micro-Plus Spirometer; Carefusion, Corp., San Diego, CA, USA) with a standard flange mouthpiece. In stroke patients with severe facial palsy and unable to perfect lip seal, the therapist aided by holding the lips around the mouthpiece minimize air leak. A live demonstration was performed by the physiotherapist and the patient was allowed to practice before the formal assessment [19]. The highest recorded values after three attempts were used for the analysis. All patients who had undergone an assessment of PCF, MIP, and MEP were included in the final analysis.

### 2.4. Clinical Variables

The post-stroke functional status was assessed using the modified Barthel Index (MBI) [29]. Berg Balance Scale (BBS), and Mini-Mental State Examination (MMSE) [30].

### 2.5. Sample Size Estimation

A power analysis was performed to determine the appropriate sample size. Taken into account that the prevalence of post-stroke dysphagia to be at 60%, a minimum sample size of 178 (including a minimum of 107 with dysphagia) was required to achieve a minimum power of 80% in order to detect a change in the sensitivity from 0.80 to 0.90, based on a target significant level of 0.05. This minimum sample size is also sufficient to detect a change in the value of specificity from 0.7 to 0.9 which will require a minimum sample of 78 subjects with 47 having the disease [31].

### 2.6. Statistical Analysis

Intergroup differences between the dys (+) group and the dys (−) group were assessed using *t*-test and chi-squared test as appropriate. Continuous variables were expressed as mean or median values and categorical variables were expressed as frequencies and percentages.

Optimal cut-off values were computed from the receiver operating characteristic (ROC) [32] curve for PCF, MIP, and MEP values against data regarding the presence of dysphagia (FOIS scores 1–5) obtained from the instrumental swallowing test. The area under the curve (AUC) values, which reflect the diagnostic accuracy and predictive ability for the presence of dysphagia, were calculated for each parameter [33]. Univariate analysis was performed to assess the risk of dysphagia based on these cut-off values. Correlation analysis among PCF, MIP, MEP, and FOIS scores was performed. A multivariable regression model was constructed using stepwise selection with entry criteria of *p* = 0.1 and stay criteria of *p* = 0.05. The discriminatory abilities of the risk prediction models were assessed using the DeLong test to evaluate the significance of increase in the AUC. Multivariable regression logistic analysis was performed to assess whether the cut-off values could predict the risk of dysphagia after other clinical variables were controlled. Statistical analyses were performed using SAS version 9.4 (SAS Institute, Cary, NC, USA) and R 2.15.3 (R Foundation for Statistical Computing, Vienna, Austria). Stata 16 (StataCorp LLC, College Station, TX, USA) was used to produce the graphs. *p*-values < 0.05 were considered statistically significant.

## 3. Results

### 3.1. Baseline Demographics

**Figure 1:**
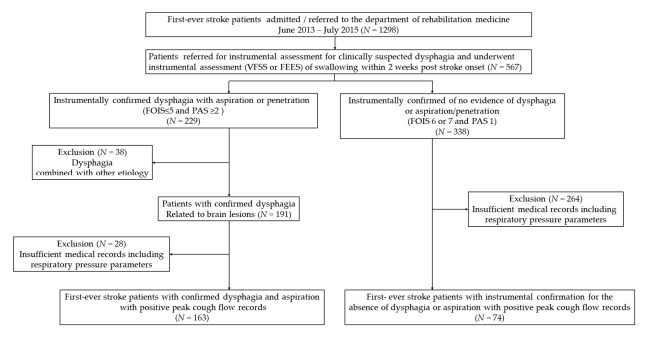
Flowchart of study participant recruitment. FEES, fiberoptic endoscopic evaluation of swallowing; VFSS, videofluoroscopic assessment of swallowing; PAS, Penetration Aspiration Scale; FOIS, Functional Oral Intake Scale.

Among 1298 admitted patients, 567 patients who complained of difficulty in swallowing or were screened to have dysphagia at initial stroke onset were referred for swallowing assessment. After the instrumental test, 338 patients were confirmed to have no evidence of dysphagia (FOIS score 6 or 7 and PAS 1). Among these, 74 patients had complete medical records of PCF and respiratory pressure parameters were classified to the dys (−) group. Among 229 patients with confirmed dysphagia after the instrumental swallowing tests, 163 met the inclusion criteria and had complete medical records were classified to the dys (+) group. Thus, medical records of 237 (female = 150, male = 87) patients were finally included (Figure 1).

There were significantly more number of patients with low body mass index in the dys (+) group and significantly more number of patients with hyperlipidemia in the dys (−) group (Table 1). No significant differences were observed in the proportion of patients with medical comorbidities including diabetes mellitus, atrial fibrillation, asthma, or chronic obstructive pulmonary disease between the groups or location of brain lesions (Supplementary Table S1).

### 3.2. Dysphagia Severity and Functional Impairment

The median time (interquartile range) interval between the day of the VFSS or FEES and the onset of cerebrovascular event related to the brain lesion was 12 (9–14) days. FEES had been performed on 45 (27.6%) patients of the dys (+) group. Patients in the dys (+) group had a median FOIS score of 2 (min-max range: 1.0–5.0) and PAS score of 7.0 (min-max range: 2.0–8.0) as confirmed by the instrumental assessment of swallowing. Patients in the dys (+) group also exhibited increased severity of other functional parameters including MBI scores and truncal control values reflected by the BBS score (Table 2).

### 3.3. PCF Results

**Figure 2:**
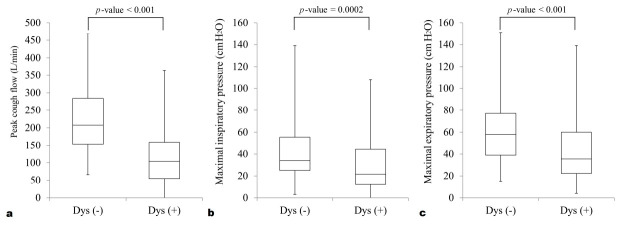
Boxplots of the (**a**) peak cough flow, (**b**) maximal inspiratory pressure and (**c**) maximal expiratory pressure. The median (interquartile range) values of the all respiratory parameters in dys (+) group are significantly smaller than those in dys (−) group. The boxplots show the medians and quartiles, and the whiskers indicate the lowest to maximum measurements.

All respiratory pressure parameters were performed within 5 days of performing the instrumental assessment of swallowing. The dys (+) group had significantly lower PCF values than the dys (−) group (116.3 ± 75.3 vs. 219.4 ± 91.8 L/min, *p* < 0.001). In addition, the dys (+) group also had lower MIP (30.5 ± 24.7 vs. 41.6 ± 25.7 cmH_2_O, *p* = 0.0002) and MEP (41.0 ± 27.9 vs. 62.8 ± 32.3 cmH_2_O, *p* < 0.001) values than the dys (−) group (Figure 2). No adverse event was recorded from participants performing the PCF meter.

### 3.4. ROC Curve Results

**Figure 3:**
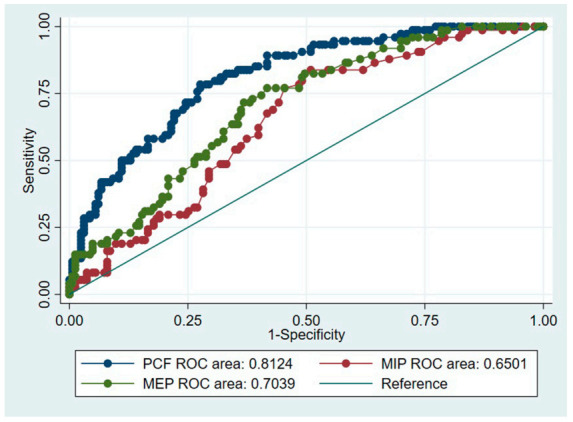
Receiver operating characteristic (ROC) curve analysis for the cut-off values measured by the peak cough flow (PCF) (L/min) and the maximal expiratory pressure (MEP) and maximal inspiratory pressure (MIP) (cmH_2_O).

ROC curve analysis revealed that the optimal cut-off value of PCF associated with presence of instrumentally confirmed dysphagia was 151 L/min (sensitivity: 0.72 [0.66–0.79], specificity: 0.78 [0.69–0.88], AUC = 0.81 [0.76–0.87]). The optimal MIP and MEP cut-off values to screen for dysphagia were 20 cmH_2_O (sensitivity: 0.49 [0.41–0.57], specificity: 0.84 [0.75–0.92], AUC = 0.65 [0.58–0.72]) and 38 cmH_2_O (sensitivity: 0.58 [0.51–0.66], specificity: 0.77 [0.67–0.87], AUC = 0.70 [0.64–0.77]), respectively (Table 3). The PCF had the highest AUC value (Figure 3).

### 3.5. Correlation Analysis

**Figure 4:**
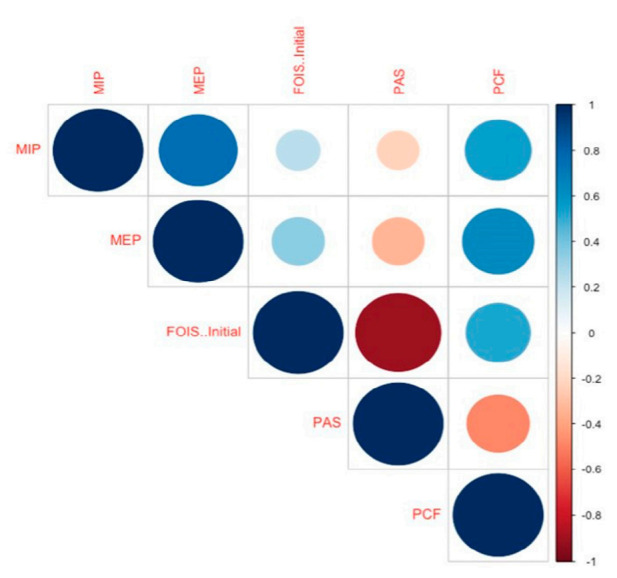
Heat map of Spearman’s correlation coefficients between the respiratory pressure parameters and the degree of dysphagia and aspiration. The color scale indicates the degree of correlation (blue: strong positive correlation, white: weak correlation, and red: strong negative correlation). FOIS, functional oral intake scale; MEP, maximal expiratory pressure; MIP, maximal inspiratory pressure; PAS, Penetration Aspiration scale; PCF, peak cough flow.

All three respiratory pressure parameters showed significant positive correlation with the FOIS score at the time of evaluation and negative correlation with the PAS score. However, the level of correlation was higher for the PCF (Spearman’s correlation coefficient: 0.508 and −0.501, respectively) than MIP (0.261 and −0.269, respectively) or MEP (0.346 and −0.361, respectively) (Figure 4). Modest degree correlation was found between the PCF and MMSE (Spearman’s correlation coefficient: 0.572).

### 3.6. Univariate Multivariable Regression Analysis

Results from the univariate analysis indicated that PCF values ≤151 L/min were associated with a 9.51-fold increase in the risk of dysphagia. MIP values ≤20 cmH_2_O and MEP values ≤38 cmH_2_O were associated with 4.98-fold and 4.68-fold increase in the risk of dysphagia, respectively.

In the final multivariable regression model that included voluntary PCF set at the cut-off value and clinical variables including low MMSE scores, the association of PCF with increased risk of dysphagia (<0.001) was maintained with adjusted odds ratio (OR) of 4.19 (2.02–83.69) and AUC value of 0.851 (0.799–0.903). After adjustment of clinical parameters, MIP and MEP set at the cut-off values showed ORs of 1.19 (0.51–2.80) and 1.23(0.56–2.68), respectively, but failed to reach statistical significance.

## 4. Discussion

The results of the present study have provided ideal cut-off values of voluntary PCF, MIP, and MEP that are associated with increased risk of dysphagia in post-stroke patients. Among these parameters, voluntary PCF (cut-off value of 151 L/min) was observed to have the highest sensitivity and specificity levels with AUC values of 0.81. PCF values below this cut-off value were associated with a 9.51-fold increase in the risk of dysphagia. This increased risk was present with an OR value of 4.19 (2.02–83.69) and an AUC value of 0.851 (0.799–0.903) even after adjustment of clinical factors including MMSE. The optimum MEP and MIP cut-off values were ≤20 cmH_2_O and ≤38 cmH_2_O, respectively. However, the AUC values (0.65 and 0.70, respectively) were low and failed to reach statistical significance in the multivariable analysis.

Our correlation analysis was in accordance with the accuracy results, with PCF showing higher correlation with the severity of dysphagia and aspiration than MIP or MEP. From an anatomical perspective, the pharyngeal muscle group responsible for nasopharynx and adductor muscle movements of the vocal cords for closing the glottis are also involved in coughing. Impaired oropharyngeal and glottic function related to dysphagia can also reduce the ability of air stacking and holding the insufflated air necessary for an effective cough. This glottic closure, which affects the compression phase of coughing may be reflected in the PCF evaluation [34] more than the MIP or MEP. In contrast, poor lip seal or inability to blow using the orofacial muscles in conjunction with weak respiratory muscles can result in reduced MIP or MEP values. However, these values do not reflect the glottic function. This might explain the higher association of PCF with dysphagia than MIP and MEP.

The PCF cut-off value identified in our study is similar to the PCF level of 160 L/min needed for extubation and tracheostomy tube removal in patients with neuromuscular dysfunction [35]. The cut-off value was lower than that reported in a recent study, which indicated that a value of 190 L/min was predictive of aspiration risk in the elderly population with community-acquired pneumonia [36]. The lower cut-off values may be related to the weakened respiratory muscles in patients with post-stroke dysphagia, which may affect the coughing force.

Cough and dysphagia are closely related, since cough, respiration, and swallowing functions share the same neural and anatomical substrates [10]. In post-stroke dysphagia, inspiratory and expiratory muscles of the oropharynx and the glottis are affected, resulting in reduced pharyngeal clearance and aspiration/penetration. Similarly, weakness of these muscles can diminish a patient’s ability to cough [6]. During a normal cough, proper glottic closure is necessary to generate a PCF with a normal flow of 360–1000 L/min. Therefore, the reduced coughing strength observed in patients with post-stroke dysphagia [2] is suggested to be a useful surrogate tool that can indirectly reflect the preservation of glottic function. Our results are not only consistent with the results of these previous studies, but also provide valid cut-off values that are associated with post-stroke dysphagia.

A recent study by Sohn et al. [19] demonstrated that a citric acid reflexive PCF cut-off of 59 L/min and a voluntary PCF cut-off of 79 L/min could aid in prediction of the risk of aspiration and respiratory complications in patients with confirmed dysphagia. However, in their study, the test had been performed in patients with dysphagia and those without dysphagia had not been included. Therefore, in addition to the cut-off values proposed by Sohn et al. [19], our study provides additional cut-off values that can help distinguish post-stroke patients with dysphagia from those without dysphagia.

Assessment of voluntary cough with the use of a spirometer is simple and its ease of application allows it to be readily performed without the need for training. Hence, voluntary PCF assessment is an ideal evaluation tool to be incorporated to assess voluntary airway clearance [37] as a part of screening procedure in current dysphagia screening protocols. The citric acid cough test shows good diagnostic properties. However, due to the need to use a nebulizer and a tussive agent [19], its daily application in the outpatient clinical settings could be technically challenging.

Disturbances in cough can be life-threatening, especially in stroke patients [38]. Cough and the ability for proper airway clearance is a vital mechanism against aspiration [39]. Early identification of patients with impaired cough who are at a higher risk of respiratory complications may be helpful. Screening of patients with dysphagia and decreased coughing function after stroke can help clinicians take preemptive measures to prevent aspiration pneumonia [11,17,40,41]. Voluntary PCF assessment is a simple and effective tool that enables effective coping with infections associated with aspiration pneumonia.

The present study has some limitations. This was a retrospective cross-sectional study, and although all parameters were assessed at the initial dysphagia assessment upon referral to our department, the clinical validity and further psychometric properties, including validity and sensitivity to change of the respiratory pressure meters need to be confirmed in future prospective studies. Moreover, future studies that incorporate more physiologic outcome measures such as swallow timing, kinematics, or airway compromise along with visualization of the glottis or cough waveform are warranted. Second, decreased alertness and cognitive function may have limited some patients’ capacity to fully cooperate with the instructions to produce a cough. To ensure that the patient had a full understanding, a pre-assessment education and practice were allowed. Moreover, Lee et al. reported that statistical correlation between the PCF and MMSE, the levels were only modest [42]. These modest levels of correlation were in parallel to our results. However, results indicate that the diagnostic parameters of the PCF are still significant; even with the adjustment of the low MMSE scores, the multivariable results showed that only the PCF was associated with dys(+) with ORs of 4.19. Such association had not been found with either of the MEP or MIP values. Low cognitive function scores can aid in the prediction of the incidence of aspiration pneumonia, allowing early assessment of oral intake capability [43]. However, our results have shown that the PCF cut-off value may still be valid for the determination of the risk of dysphagia even after adjustment of these low cognitive scores. Future studies that explore the relationship between the voluntary PCF in those with lower levels of cognition are warranted. Another point to take into consideration is the moderate sensitivity levels of the PCF. Although the voluntary PCF showed higher levels of sensitivity levels than the MIP or MEP, these values were comparable [44,45] ore even lower than other current swallowing screening tests [33,46] albeit higher specificity levels. Though high sensitivity levels to accurate screen those with risk of dysphagia is crucial, high specificity levels are important because high false positive rates may lead to unnecessary restrictions of oral intake [47]. Though in need of future prospective studies, one may cautiously postulate that the relativity higher specificity levels of the PCF may help complement the diagnostic parameters of current screening tests when used in parallel and lead to a higher net specificity and overall accuracy levels [48].

## 5. Conclusions

The results of this study indicated that among the three respiratory pressure parameters under study, voluntary PCF showed favorable AUC values that were linked with increased risk of post-stroke dysphagia. In clinical context, these values may be used as a supplementary test or may be incorporated into other standard screening tools for dysphagia [43]. It is unclear whether the performance level of current dysphagia screening tests [12,13,14,43] could be improved with the aid of PCF cut-off values and whether these values could be used as markers of improved respiratory function along with post-stroke respiratory training [11]. These topics need to be pursued in future studies. Nevertheless, the ideal cut-off values provided in our study may help complement screening tests for patients with post-stroke dysphagia and help identify those who may have impaired cough and respiratory function.

## Figures and Tables

**Table 1:** Basic demographic characteristics of the participants.

Variables	Dys (−) (*n* = 74)	Dys (+) (*n* = 163)	*p*
Age (years)	61.3 ± 12.3	63.8 ± 14.7	0.113
Body mass index (kg/m^2^)	22.4 (22.0–25.0)	22.1 (20.0–24.5)	0.072
<18.5	2 (8.7)	21 (91.3)	0.043 *
18.5–24.9	53 (32.7)	109 (67.3)	
≥25	19 (36.5)	33 (63.5)	
Gender			
Women	48 (64.9)	102 (62.6)	0.735
Men	26 (35.1)	61 (37.4)	
Medical comorbidities			
Smoking	33 (32.4)	69 (67.6)	0.744
Chronic obstructive pulmonary disease	0 (0.0)	4 (100.0)	0.313
Asthma	0 (0.0)	3 (100.0)	0.554
Diabetes mellitus	16 (22.5)	55 (77.5)	0.059
Atrial fibrillation	10 (32.3)	21 (67.7)	0.894
Hypertension	42 (31.3)	92 (68.7)	0.964
Hyperlipidemia	10 (58.8)	7 (41.2)	0.011 *
Coronary artery disease	2 (20.0)	8 (80.0)	0.729

Values are numbers (percentages) for categorical variables and means ± standard deviation or median (range) for others. *p*-values were determined by using the chi-square, Fisher’s exact, or Wilcoxon rank sum test. * *p* < 0.05.

**Table 2:** Comparison of the clinical variables between those who were diagnosed with dysphagia versus those with no dysphagia.

	Dys (+) (*n* = 163)	Dys (−) (*n* = 74)	*p*
Functional parameters			
Modified Barthel Index	31.9 ± 31.5	60.1 ± 31.6	0.0084
Berg Balance Scale	19.2 ± 22.0	31.0 ± 21.8	<0.001
MMSE	17.0 (9.0–24.0)	26.0 (24.0–28.0)	<0.001
Initial dysphagia severity			
PAS	7.0 (6.0–8.0)	1.0 (1.0–1.0)	<0.001
FOIS	2.0 (1.0–4.0)	7.0 (6.0–7.0)	<0.001

Values are means (standard deviation) or median (interquartile range). *p*-values were determined by using the chi-square, Fisher’s exact test, or Wilcoxon rank sum test. MMSE, Mini-Mental State Examination; PAS, Penetration Aspiration Scale; FOIS, Functional Oral Intake Scale.

**Table 3:** Diagnostic parameters of the optimal cut-off points of the respiratory pressure meters to diagnose presence of dysphagia defined as FOIS levels 1–5.

Variables	Dys (−)	Dys (+)	Sensitivity	Specificity	PPV	NPV
PCF (L/min)						
>151	58	45	0.72 (0.66–0.79)	0.78 (0.69–0.88)	0.88 (0.83–0.94)	0.56 (0.47–0.66)
≤151	16	118				
MIP (cmH_2_O)						
>20	62	83	0.49 (0.41–0.57)	0.84 (0.75–0.92)	0.87 (0.80–0.94)	0.43 (0.35–0.51)
≤20	12	80				
MEP (cmH_2_O)						
>38	62	83	0.58 (0.51–0.66)	0.77 (0.67–0.87)	0.85 (0.78–0.91)	0.46 (0.37–0.54)
≤38	12	80				

Cut-off value determined using ROC curve analysis (with youden index). Results presented as values (95% confidence intervals). FOIS, Functional Oral Intake Scale; PPV, positive predictive value; NPV, negative predictive value; PCF, peak cough flow; MEP, maximal expiratory pressure; MIP, maximal inspiratory pressure.

## Supplementary Materials

The following are available online at https://www.mdpi.com/1010-660X/56/12/635/s1, Table S1: Basic characteristics of the brain lesions of the participants.
