# Supplementary material for: Cut-off Values of the Respiratory Muscle Power and Peak Cough Flow in Post-Stroke Dysphagia
Source: Medicina (Kaunas). 2020 Nov 24;56(12):635. doi: 10.3390/medicina56120635 (PMC7760136; doi:10.3390/medicina56120635)
Supplement: Supplementary file 1 [file medicina-56-00635-s001.pdf]

Supplemental Table 1 Basic characteristics of the brain lesions of the participants

Variables	Dys(-)<br>(n = 74)	Dys(+)<br>(n = 163)	*P*
Laterality			
Right	34 (36.6)	59 (63.4)	0.154
Left	15 (18.8)	65 (81.3)	0.003
Bilateral	7 (19.4)	29 (80.6)	0.098
Multiple	18 (64.3)	10 (35.7)	<0.001
Etiology type			
Ischemic	31 (24.6)	95 (75.4)	0.0019
Intracerebral hemorrhage	16 (31.4)	35 (68.6)	0.979
Subarachnoid hemorrhage	4 (19.0)	17 (81.0)	0.207
Traumatic brain injury	13 (54.2)	11 (45.8)	0.011
Others[a]	10 (66.7)	5 (33.3)	0.002
Location			
Cortical	17 (22.7)	58 (77.3)	0.061
Subcortical	23 (29.5)	55 (70.5)	0.736
Infratentorial	12 (26.1)	34 (73.9)	0.428
Multiple	2 (33.3)	4 (66.7)	0.897
Others[b]	19 (61.3)	12 (38.7)	<0.001

Values are numbers (percentages) for categorical variables and means (standard deviation) or median (range) for others.

P-values were determined by using the chi-square, Fisher's exact test, or Wilcoxon rank sum test.

[a]Brain neoplasm, hydrocephalus.

[b]Diffuse axonal injury, intraventicular, subarachnoid space.
